# Difference in *agr* Dysfunction and Reduced Vancomycin Susceptibility between MRSA Bacteremia Involving SCC*mec* Types IV/IVa and I–III

**DOI:** 10.1371/journal.pone.0049136

**Published:** 2012-11-12

**Authors:** Hee-Chang Jang, Seung-Ji Kang, Su-Mi Choi, Kyung-Hwa Park, Jong-Hee Shin, Hyon E. Choy, Sook-In Jung, Hong Bin Kim

**Affiliations:** 1 Department of Infectious Diseases, Chonnam National University Medical School, Gwang-ju, Republic of Korea; 2 Department of Laboratory Medicine, Chonnam National University Medical School, Gwang-ju, Republic of Korea; 3 Department of Microbiology, Chonnam National University Medical School, Gwang-ju, Republic of Korea; 4 Department of Internal Medicine, Seoul National University College of Medicine, Seoul, Republic of Korea; National Institutes of Health, United States of America

## Abstract

**Background:**

Dysfunction of *agr*, with reduced susceptibility or hetero-resistance to vancomycin, is thought to be associated with a worse outcome of methicillin-resistant *Staphylococcus aureus* (MRSA) bacteremia (MRSAB). However, the difference in *agr* dysfunction according to the SCC*mec* type in MRSA infection is undetermined. We compared the prevalence of *agr* dysfunction, reduced vancomycin susceptibility and the outcomes of SCC*mec* IV/IVa and I–III MRSAB.

**Methods:**

The study included 307 cases of MRSAB. SCC*mec* types were determined by multiplex PCR. The clinical and microbiological features and outcomes of 58 SCC*mec* IV/IVa MRSAB were compared with those of 249 SCC*mec* I–III MRSAB.

**Results:**

Compared with SCC*mec* I–III MRSAB, SCC*mec* IV/IVa MRSAB was associated with lower rates of *agr* dysfunction (3% *vs*. 43%), vancomycin minimum inhibitory concentration (MIC) = 2 µg/mL (3% *vs*. 15%), and hetero-resistance to vancomycin (0% *vs*. 8%) (all *P*<0.05). However, the 30-day and *S*. *aureus*-related mortality in patients with SCC*mec* IV/IVa MRSAB were not different from those in patients with SCC*mec* I–III MRSAB in multivariate analyses (HR 1.168, 95% CI 0.705–1.938; HR 1.025, 95% CI 0.556–1.889).

**Conclusions:**

SCC*mec* IV/IVa MRSAB was associated with lower rates of *agr* dysfunction and hetero-resistance to vancomycin and a lower vancomycin MIC, compared with SCC*mec* I–III MRSAB. However, the outcomes of SCC*mec* IV/IVa MRSAB did not differ from those of SCC*mec* I–III MRSAB.

## Introduction

Accessory gene regulator (*agr*) is a global regulator gene of *Staphylococcus aureus* that controls the expression of major virulence factors, such as cytotoxins, enzymes, and superantigens [1]. Moreover, *agr* is the main quorum-sensing operon in *S. aureus* that regulates cell to cell signaling [2], [3], Traditionally, most human *S. aureus* isolates are considered *agr^+^* and to have *agr* function; however, *S. aureus* with diminished or absent δ-hemolysin expression (*agr* dysfunction), the end-product of the *agr* system, has recently emerged and become prevalent in methicillin-resistant *S. aureus* (MRSA) [4].

Dysfunction of *agr* is thought to be associated with decreased susceptibility to vancomycin and vancomycin-intermediate *S*. *aureus* (VISA)/hetero-VISA [5]–[7], and some have suggested that *agr* dysfunction adversely affects the treatment outcomes of MRSA infection [8]. However, the prevalence of *agr* dysfunction according to the SCC*mec* type in MRSA infection remains uncertain, although MRSA possessing SCC*mec* type IV/IVa (SCC*mec* type IV/IVa MRSA), known as a community-associated MRSA clone, has different antibiotic susceptibility patterns and toxin profiles from MRSA possessing SCC*mec* types I–III (SCC*mec* I–III MRSA). Moreover, it is still not known whether the outcomes of bacteremia caused by SCC*mec* IV/IVa MRSA (SCC*mec* IV/IVa MRSAB) are similar to that caused by SCC*mec* I–III MRSA (SCC*mec* I–III MRSAB), because clinical studies have obtained conflicting results [9]–[14].

This study compared the prevalence of *agr* dysfunction, hetero-VISA, and the vancomycin minimum inhibitory concentration (MIC) of SCC*mec* IV/IVa MRSAB with those of SCC*mec* I–III MRSAB, and investigated the impact of these factors on the outcomes of MRSA bacteremia.

## Patients and Methods

### Ethics

This Study was approved by the institutional review board of Chonnam National University Hospital. A waiver of consent was granted given the retrospective nature of the project.

### Patients

All patients ≥16 years old with MRSA bacteremia who were treated between January 2005 and December 2008 at two university hospitals and referral center centers, Chonnam National University Hospital (1000 beds; Gwang-ju, Republic of Korea) and Chonnam National University Hwasun Hospital (700 beds; Hwasun, Republic of Korea), were included. Cases were identified using computerized records from the Clinical Microbiology Laboratory. Only the first episode of MRSA bacteremia in a patient was included. Demographic and clinical data were collected by reviewing the electronic medical records of the patients.

### Microbiological Tests

*S. aureus* was identified and methicillin resistance was determined using the automated systems Vitek 2 (bioMérieux, Marcy l’Etoile, France) or Microscan (Dade Behring Inc., Deerfield, IL). MICs of vancomycin were determined by Etest (AB BIODISK, Solna, Sweden) using a 0.5 McFarland inoculum on Muller–Hinton agar plates. Modified population analyses for hetero-VISA detection were performed using brain–heart infusion agar (BHIA; BD Diagnostics, Sparks, MD) plates containing various concentrations of vancomycin [15]. ATCC 29213, Mu50 (a VISA strain), and Mu3 (a hetero-VISA strain) were used as controls for Etest and modified population analysis. *agr* dysfunction was determined by examining δ-hemolysin expression on blood agar plates using *S. aureus* RN4220, as described previously [6].

Multiplex PCR was performed to determine SCC*mec* type for MRSA isolates, as described previously [16]–[19]. Panton–Valentine leukocidin (*pvl*) genes were detected by PCR, as described previously [20].

### Definitions

*S. aureus* bacteremia was considered to have been *hospital-onset* if *S. aureus* was isolated from cultures of blood samples obtained from patients who had been hospitalized for 48 h or longer. Otherwise, *S. aureus* bacteremia was considered to have been *community-onset*. *S. aureus* bacteremia was defined as *community-acquired* if *S. aureus* were isolated from cultures of blood samples obtained within 48 h of hospital admission and the patient had no medical history of MRSA infection or colonization. This included no medical history in the past year of dialysis, surgery, hospitalization, admission to a nursing home, skilled nursing facility, or hospice, and no permanent indwelling catheter or medical device that passed through the skin into the body [21]. Otherwise, *S. aureus* bacteremia was considered to have been *health care-acquired*.

*S. aureus* bacteremia was defined as *catheter-related* if the catheter tip grew more than 15 colonies for *S. aureus*, or inflammation was present at the insertion site and no alternative source of infection was identified [22]. *Infective endocarditis* was defined by the modified Duke criteria [23]. *Metastatic infection* was defined as the presence of microbiological or radiographic evidence of *S. aureus* infection caused by hematogenous seeding [22]. *Persistent bacteremia* was defined as consecutive blood cultures positive for 7 or more days despite appropriate antibiotic use for 5 or more days [24]. Mortality was defined as *S. aureus-related* in the absence of another definite cause of death [24].

### Statistical Analyses

Categorical variables were compared using Fisher’s exact test or the Pearson χ^2^ test as appropriate, and continuous variables were compared using Student’s *t-*test. Multivariate analyses were performed using the Cox-regression hazard model in the backward stepwise conditional manner. All tests of significance were two-tailed, and *P* values ≤0.05 were deemed to indicate statistical significance. Statistical analyses of the data were performed using the PASW statistics software (version 18.0; SPSS Inc., Chicago, IL).

## Results

### SCC*mec* Type and pvl in MRSA Blood Isolates

We identified 307 cases of first-episode MRSA bacteremia during the study period. The most common SCC*mec* type was II (67.4%) followed by III (13.4%), IVa (12.4%), and IV (6.5%). Only one SCC*mec* type IVa isolate carried *pvl.* The prevalence of *agr* dysfunction and the MICs of vancomycin were significantly lower in SCC*mec* IV/IVa MRSA than SCC*mec* I–III MRSA (*P*≤0.05, each; Table 1). Hetero-VISA was observed only in SCC*mec* I–III MRSA clones (Table 1). SCC*mec* type IV/IVa isolates presented lower resistance rates to non-β-lactam antibiotic agents (*P*≤0.05, each; Table 1).

**Table 1:** Microbiologic characteristics of 307 MRSA bacteremic isolates according to the SCC*mec* type.

Characteristics	SCC*mec* type^a^							*P* value^b^
	I(n = 1)	II(n = 207)	III(n = 41)	IV(n = 20)	IVa(n = 38)	I–III(n = 249)	IV/IVa(n = 58)	
*agr* dysfunction	1 (100)	84 (41)	21 (51)	1 (5)	1 (3)	106 (43)	2 (3)	<0.001
hetero-VISA	0 (0)	15 (7)	4 (10)	0 (0)	0 (0)	19 (8)	0 (0)	0.030
Vancomycin MIC								
≤1 µg/mL	0 (0)	93 (45)	7 (17)	19 (95)	31 (82)	100 (40)	50 (86)	<0.001
1.5 µg/mL	0 (0)	88 (43)	24 (59)	1 (5)	5 (13)	112 (45)	6 (10)	
2 µg/mL	1 (100)	26 (13)	10 (24)	0 (0)	2 (5)	37 (15)	2 (3)	
Antimicrobial susceptibility								
Clindamycin	0 (0)	9 (4)	2 (5)	14 (70)	34 (90)	11 (4)	48 (83)	<0.001
Erythromycin	0 (0)	4 (2)	0 (0)	12 (60)	29 (76)	4 (2)	41 (71)	<0.001
Ciprofloxacin	0 (0)	22 (11)	0 (0)	14 (70)	37 (97)	22 (9)	51 (88)	<0.001
Gentamicin	0 (0)	36 (17)	0 (0)	14 (70)	34 (90)	36 (14)	48 (83)	<0.001
TMP/SMX	1 (100)	195 (94)	10 (24)	19 (95)	38 (100)	206 (83)	57 (98)	0.002

**NOTE**. hetero-VISA, hetero-vancomycin-intermediate *S. aureus*; MIC, minimum inhibitory concentration; TMP/SMX, trimethoprim/sulfamethoxazole.

a Results represent number with the percentage indicated in parentheses unless otherwise specified.

b Comparison of SCC*mec* I–III MRSA with SCC*mec* IV/IVa MRSA.

### Clinical Features and Outcome of SCC*mec* IV/IVa MRSAB as Compared with SCC*mec* I–III MRSAB

The clinical features of SCC*mec* IV/IVa MRSAB and SCC*mec* I–III MRSAB are shown in Table 2. SCC*mec* IV/IVa MRSAB was significantly more associated with community-acquired and community-onset infection than SCC*mec* I–III MRSAB (*P*≤0.05, each). Skin and soft-tissue infections (SSTIs) were significantly more common; however, vascular catheter-related infection was significantly less common in SCC*mec* IV/IVa MRSAB compared with SCC*mec* I–III MRSAB (*P*≤0.05, each). Metastatic infection was more commonly observed in SCC*mec* IV/IVa MRSAB than in SCC*mec* I–III MRSAB (*P*≤0.05). However, APACHE II score did not differ statistically between two groups (*P* = 0.729). The use of glycopeptides as a definitive therapy of MRSAB was more common in SCC*mec* I–III MRSAB than SCC*mec* IV/IVa MRSAB (*P* = 0.004).

**Table 2:** Clinical features of 307 patients with SCC*mec* IV/IVa MRSAB or SCC*mec* I–III MRSAB.

Characteristics	No.(%) of patients with		*P* value
	SCC*mec* IV/IVaMRSAB(n = 58)	SCC*mec* I–IIIMRSAB(n = 249)	
Age^a^	62.0±15.1	59.5±15.6	0.280
Acquisition			
Community-onset	17 (30)	31 (12)	0.001^b^
Community-acquired	5 (9)	6 (2)	0.038^b^
Underlying disorder			
Diabetes	21 (36)	76 (31)	0.402
Cancer	16 (28)	29 (12)	0.002^b^
Cerebrovascular accident	8 (14)	57 (23)	0.127
Liver cirrhosis	6 (10)	23 (9)	0.795
Congestive heart failure	6 (10)	24 (10)	0.873
Renal replacement therapy	5 (9)	23 (9)	0.883
Chronic obstructive lung disease	2 (3)	16 (6)	0.542
APACHE II score^a^	19.5±10.4	19.9±8.9	0.729
Primary site of infection			
Skin and soft tissue	22 (38)	43 (17)	0.001^b^
Bone and joint	8 (14)	18 (7)	0.118
Intravascular catheter	10 (17)	91 (37)	0.005^b^
Lung	6 (10)	23 (9)	0.795
Intra-abdominal	2 (3)	21 (8)	0.271
Complicated bacteremia			
Infective endocarditis	0 (0)	2 (1)	>0.999
Persistent bacteremia	9 (16)	19 (8)	0.060
Metastatic infection	11 (19)	6 (2)	<0.001^b^
Therapy			
Adequate empirical antibiotics within 48 h	26 (45)	91 (37)	0.242
Glycopeptides as definitive antibiotics	35 (60)	196 (79)	0.004^b^
Eradication of infection foci	21 (36)	96 (39)	0.740
Outcomes			
30-day crude mortality^c^	20/58 (35)	78/245 (32)	0.698
30-day *S. aureus*-related mortality^c^	18/58 (31)	58/245 (24)	0.245

**NOTE**. SCC*mec* IV/IVa MRSAB, bacteremia caused by MRSA possessing SCC*mec* type IV or IVa; SCC*mec* I–III MRSAB, bacteremia caused by MRSA possessing SCC*mec* types I–III; APACHE, acute physiology and chronic health evaluation.

a Continuous variables are expressed as means (±SD).

b Statistically significant (*P*≤0.05).

c Expressed as number of deaths/number of patients followed up (%).

### Outcomes of SCC*mec* IV/IVa MRSAB Compared with SCC*mec* I–III MRSAB

Univariate and multivariate analysis for risk factors associated with 30-day mortality in patients with MRSAB are shown in Table 3.In the univariate analysis, age, cancer, chronic obstructive lung disease, and APACHE II score were all significantly associated with increased mortality; but eradication of infection foci was negatively related to 30-day mortality (*P*≤0.05, each). Increased vancomycin MIC (2 µg/mL), hetero-VISA, and *agr* dysfunction were not associated with increased 30-day mortality in the univariate analysis. In the multivariate analysis, cancer and APACHE II scores were independent risk factors for-30 day mortality, and the eradication of infective foci was negatively related to 30-day mortality in patients with MRSAB.

**Table 3:** Univariate and Multivariate analyses for risk factors associated with 30-day mortality in patients with MRSA bacteremia.

	Univariate analysis			Multivariate analysis			
Risk Factor	No.(%) of patients		*p*-value	HR	95% CI		*p*-value
	Survival(n = 209)	Death(n = 98)			Lower	Upper	
Age^a^	58.6±16.0	63.1±14.1	0.017^b^				
Acquisition							
Hospital-onset	178 (85)	81 (83)	0.572				
Health care-acquired	203 (97)	93 (95)	0.327				
Underlying diseases							
Diabetes	63 (30)	34 (35)	0.424				
Cancer	24 (12)	21 (21)	0.022^b^	2.026	1.228	3.343	0.006^b^
Liver cirrhosis	19 (9)	10 (10)	0.756				
Renal replacement therapy	20 (10)	8 (8)	0.690				
Congestive heart failure	18 (9)	12 (12)	0.324				
Cerebrovascular accident	44 (21)	21 (21)	0.940				
Chronic obstructive lung disease	7 (3)	11 (11)	0.006^b^				
Primary site of infection							
Skin and soft tissue	46 (22)	19 (19)	0.600				
Bone and joint	19 (9)	7 (7)	0.568				
Lung	16 (8)	13 (13)	0.117				
Intravascular catheter-related	74 (35)	27 (28)	0.172				
Intra-abdominal infection	18 (0)	5 (5)	0.276				
Primary bacteremia	38 (18)	27 (28)	0.061				
Complicated bacteremia							
Infective endocarditis	1 (0)	1 (1)	0.537				
Other metastatic infection	9 (4)	8 (8)	0.168				
Persistent bacteremia	16 (8)	12 (12)	0.193				
APACHE II score^a^	16.5±6.9	27.0±9.3	<0.001^b^	1.127	1.102	1.152	<0.001^b^
Treatment							
Adequate antibiotics within 48 hours	81 (39)	36 (37)	0.734				
Glycopeptides as definitive antibiotics	163 (78)	68 (69)	0.104				
Eradication of infection foci	95 (46)	22 (22)	<0.001^b^	0.575	0.349	0.950	0.031^b^
Microbiological characteristics							
SCC*mec* IV/IVa MRSA	38 (18)	20 (20)	0.642				
hetero-VISA	13 (6)	6 (6)	0.974				
* agr* dysfunction	72 (34)	36 (37)	0.696				
Vancomycin MIC = 2 µg/mL	27 (13)	12 (12)	0.869				

**NOTE**. HR, hazard ratio; CI, confidence interval; APACHE, acute physiology and chronic health evaluation; SCC*mec* IV/IVa MRSA, MRSA possessing SCC*mec* type IV or IVa; hetero-VISA, hetero-vancomycin-intermediate *S. aureus*; MIC, minimum inhibitory concentration.

a Continuous variables are expressed as means (±SD).

b Statistically significant (*P*≤0.05).

**Figure 1:**
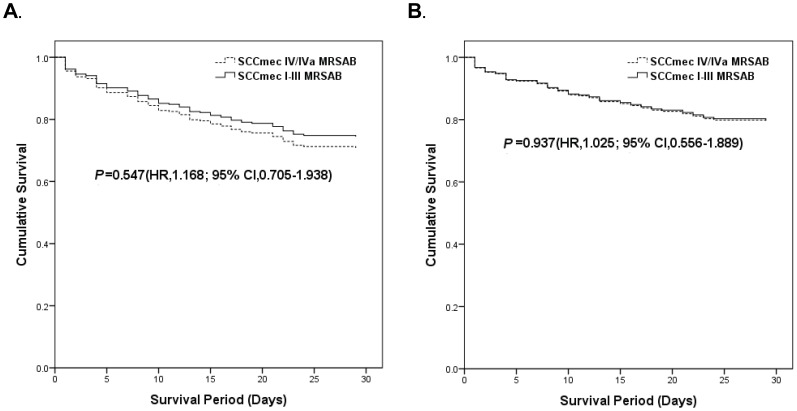
Adjusted 30-day crude and 30-day *S. aureus*-related mortalities in patients with SCC*mec* IV/IVa MRSAB or SCC*mec* I–III MRSAB. A. Adjusted 30-day mortalities in patients with SCC*mec* IV/IVa MRSAB or SCC*mec* I–III MRSAB by multivariate Cox-regression survival analysis. B. Adjusted 30-day *S. aureus*-related mortalities in patients with SCC*mec* IV/IVa MRSAB or SCC*mec* I–III MRSAB by multivariate Cox-regression survival analysis. NOTE. SCC*mec* IV/IVa MRSAB, bacteremia caused by MRSA possessing SCC*mec* type IV or IVa; SCC*mec* type I–III MRSAB, bacteremia caused by MRSA possessing SCC*mec* types I–III.

Thirty-day crude and 30-day *S. aureus*-related mortalities were not significantly different between patients with SCC*mec* IV/IVa MRSAB and those with SCC*mec* I–III MRSAB (Table 2, Fig. 1).Thirty-day crude and 30-day *S. aureus*-related mortalities also did not differ between patients with SCC*mec* IV/IVa MRSAB and SCC*mec* I–III MRSAB in multivariate analyses, despite adjustment of independent risk factors using a Cox-regression model (Fig. 1).

## Discussion

In the present study, we found that SCC*mec* IV/IVa MRSA were associated with low rates of *agr* dysfunction, compared with SCC*mec* I–III MRSA. However, outcomes of SCC*mec* IV/IVa MRSAB were not different from those of SCC*mec* I–III MRSAB.

Although *agr* dysfunction was suggested as contributing to increased mortality related to *S. aureus* bacteremia, little is known of the prevalence in CA-MRSA clones possessing SCC*mec* type IV/IVa as compared with HA-MRSA clones possessing SCC*mec* type I–III. In our previous study, the frequency of *agr* dysfunction in MSSA blood isolates was 14% and this rate was significantly lower than that in MRSA isolates [25]. In this study, we found similar results; the prevalence of *agr* dysfunction was significantly lower in SCC*mec* IV/IVa MRSA than SCC*mec* I–III MRSA. SCC*mec* IV/IVa MRSA clones were more similar to MSSA than SCC*mec* I–III MRSA clones in terms of the prevalence of *agr* dysfunction.

Previous studies demonstrated a limited vancomycin resistance potential in SCC*mec* IV/IVa MRSA clones [26], [27]. However, recently, a SCC*mec* IV/IVa MRSA clone with an hetero-VISA or VISA phenotype was described [28]–[30], suggesting that hetero-VISA is not limited to typical ‘hospital’ clones of *S. aureus*. Han *et al.* [31] recently showed that the reduced vancomycin susceptibility was lower in SCC*mec* IV MRSA blood isolates than SCC*mec* II MRSA isolates, in concordance with the current study. However, the prevalence of hetero-VISA and *agr* dysfunction of SCCmec IV MRSA isolates were not directly compared with those of SCC*mec* II MRSA isolates in that study. In this study, the hetero-VISA phenotype developed only in SCC*mec* I–III MRSA and vancomycin MICs were significantly lower in SCC*mec* IV/IVa MRSA. Our data suggest that although hetero-VISA or MRSA with vancomycin MIC = 2 µg/mL can be found in all MRSA lineages, their prevalence was still significantly lower in SCC*mec* IV/IVa MRSA.

In this study, SSTI was significantly more common; however, vascular catheter-related infection was significantly less common in SCC*mec* IV/IVa MRSAB compared with SCC*mec* I–III MRSAB. Some investigators have shown that the *agr* system and α-hemolysin play essential roles in pathogenesis of *S. aureus* SSTI [32], [33] in animal models. However, these roles have not been evaluated in human diseases. Our observational clinical findings regarding the association between SCC*mec* IV/IVa MRSA, which expresses *agr* and α-hemolysin, with SSTI in human disease consistently provide further evidence of the important role of the *agr* system and α-hemolysin in the pathogenesis of *S. aureus* SSTI. Although *agr* positively regulates cytotoxins and enzymes, it negatively regulates the biofilm-producing ability of *S. aureus* [2], [34] and biofilm-producing ability of *agr*-dysfunctional MRSA blood isolates are higher compared to *agr*-functional MRSA blood isolates in our previous study [25]. SCC*mec* I–III MRSA showing high rate of *agr* dysfunction was a more common cause of catheter-related infection than SCC*mec* IV/IVa MRSA in this study. These findings suggest that the higher biofilm-producing ability of *agr*-dysfunctional MRSA might contribute to catheter-colonization and subsequent catheter-related infections, compared to *agr*-functional MRSA.

The outcomes of MRSA bacteremia are poorer than those of MSSA bacteremia [35]. However, studies on the outcomes of SCC*mec* IV/IVa MRSAB as compared with SCC*mec* I–III MRSAB show conflicting results. Chen *et al*. reported that mortalities in patients with SCC*mec* IV/IVa MRSAB were significantly lower in SCC*mec* I–III MRSAB [9]. However, these results were derived only from selected patients (those with community-onset bacteremia in the emergency department) and used 90-day mortality (instead of the more commonly applied 30-day mortality) as an outcome measure, which can be more affected by underlying conditions than *S. aureus* bacteremia itself. Note that in another study performed by the same group, the 14- and 30-day mortalities were not significantly different between patients with nosocomial SCC*mec* IV/IVa MRSAB and SCC*mec* I–III MRSAB [14], as well as data from the current study and those of another group [10]–[12].

We initially hypothesized that SCC*mec* IV/IVa MRSAB was associated with better outcomes than SCC*mec* I–III MRSAB because we thought SCC*mec* IV/IVa MRSA might be associated with lower rates of *agr* dysfunction and hetero-VISA phenotype and decreased vancomycin MICs than SCC*mec* I–III MRSAB clones. A recent study suggested that *agr* dysfunction was associated with higher mortality in MRSA bacteremia [8], and some data show an association between vancomycin MICs and the hetero-VISA phenotype and higher mortality rates [36]–[38]. However, in this study, the mortality rate in patients with SCC*mec* IV/IVa MRSAB was not different from that in patients with SCC*mec* I–III MRSAB, even though SCC*mec* IV/IVa MRSA clones had lower rates of *agr* dysfunction, hetero-VISA, and lower vancomycin MICs. In this study, *agr* dysfunction was not associated with increased mortality in MRSA bacteremia, in contrast to a previous report [8]. Neither vancomycin MICs nor the hetero-VISA phenotype was associated with higher mortality rates in this study, in agreement with previous reports [39]–[46].

Two possible explanations exist for this result. One is that *agr* dysfunction, vancomycin MICs, and the hetero-VISA phenotype did not themselves adversely influence the outcome of MRSA bacteremia in vivo. The second is that the virulence attenuation caused by *agr* dysfunction might compromise the adverse influence on mortality of decreased sensitivity to glycopeptides in patients with MRSA bacteremia. Peleg *et al*. showed that in MRSA with *agr* dysfunction that had developed increased vancomycin MIC and the hetero-VISA/VISA phenotype, virulence toward *Galleria mellonella* was attenuated [47]. This latter hypothesis might be supported by the findings of other clinical studies: the paradoxical relationship between increased vancomycin MIC and the decreased mortality and septic shock rates in MRSA bacteremia [37], [41], [44], and the similar outcomes of SCC*mec* IV/IVa MRSAB and SCC*mec* I–III MRSAB, despite the high prevalence of both complicated (this study) and severe infections in SCC*mec* IV/IVa MRSAB [10], [11].

Our study has some limitations. First, only one MRSA isolate included in this study possessed *pvl.* For this reason, our results are limited to *pvl*-negative SCC*mec* IV/IVa MRSA clones. Further investigation is needed, including more common SCC*mec* IV/IVa MRSA clones such as US300. Second, serum glycopeptide levels could affect the outcomes of SAB and act as a confounding factor, but these values were not included in the analysis because serum vancomycin levels were not measured in all patients. Third, because only one isolate per patient was examined, there is some possibility that the results may not reflect the *agr* status of all the bloodstream MRSA population but only reflect the predominant population within each patient. Fourth, only *agr* status, not the overall virulence gene expression of the individual strains, was examined in this study.

In conclusion, the rates of *agr* dysfunction, hetero-VISA phenotype, and increased vancomycin MICs were lower in SCC*mec* IV/IVa MRSAB than in SCC*mec* I–III MRSAB in this study. However, the outcomes of SCC*mec* IV/IVa MRSAB did not differ from those of SCC*mec* I–III MRSAB.
